# The professional contribution of chiropractors to Danish elite football clubs: a qualitative exploration of role and perceived value in an interprofessional service provision context

**DOI:** 10.1186/s12998-020-00358-x

**Published:** 2020-12-18

**Authors:** Joachim Hostrup, Anders Koza, Corrie Myburgh

**Affiliations:** Department of Sports Science and Clinical Biomechanics, University of Southern Denmark, 5230 Odense M, Denmark

**Keywords:** Sports chiropractic, Sports medicine, Interprofessional practice, Healthcare team

## Abstract

**Background:**

Interprofessional team-based care has been widely adopted in elite level athletic health and performance practice. Chiropractors can claim some penetration as health care service providers in high level sport. However, their position as valued members of interprofessional health care teams, especially those built around traditional medical organisational structures, is unclear. This investigation sought to explore the perceived role and value of chiropractors as service providers in elite Danish football clubs.

**Methods:**

A comparative qualitative case study was conducted. Six Danish premiere league (Superliga) clubs were purposively sampled to compare and contrast instances where chiropractors were both present and absent from the health care team. Triangulated responses were solicited from healthcare coordinators, chiropractors and athletes within each club’s organization through semi-structured individual interviews. The audio-recorded responses were transcribed verbatim and thematically analyzed using a framework approach.

**Results:**

Data were collected September and November 2019. A coding framework of 14 codes and 4 code families emerged, centering around the role of chiropractors, benefits of utilizing chiropractic care and facilitators and barriers to interprofessional practice. From this framework, three themes were abstracted, these being: “Broadening horizons”, “In-house preferred to take-away” and “Already covered, or even necessary?”

**Conclusion:**

In this practice context, chiropractors fill the role of musculoskeletal health care service providers. Their perceived value stems from additional expert disciplinary knowledge, improved diagnostic triage and increased treatment flexibility. However, where not utilized, the role of a spinal health expert is questioned and when acknowledged, is limited to that of a technician/therapist. It is unclear from this investigation whether chiropractors can claim core provider status. Further exploration of this interesting context of interprofessional practice is warranted.

## Background

Interprofessional health care practice occurs when practitioners from different backgrounds combine their unique disciplinary knowledge, in an effort to provide a superior quality of service to people living with a particular health care challenge [1, 2]. Interprofessional practice has progressively gained support as an innovative approach to coping with future health care delivery demands, and significant efforts have been made to both increase and optimize its occurrence [3–5].

In the world of high stakes elite and professional sport, organizations gamble heavily on identifying potential ‘X factor’ athletes, in the hopes of realizating performance goals and/or profitability [6]. Sidelined athletes give a poor return on investment, and therefore in instances of athletic injury, rapid return-to-play is prioritized [7]. With this strategic goal in mind, sporting organizations now commonly draw expertise from different professions into interprofessional health care teams (IPHCT) [8].

The perpetual drive for a establishing/maintaining a competitive edge over other sporting teams appears to extend into health service delivery. As a consequence, non-traditional occupational groups with perceived value, have an opportunity to find inclusion in the IPHCT [8]. This context therefore extends interprofessional practice beyond that of ordinary (mainstream) health care providers to include professions classified as so-called complementary and alternative medicine (CAM) provider groups.

Chiropractors are frequently classified as CAM service providers and claim a role as service providers in elite level athletic health and performance [9, 10]. Generally speaking, their inclusion is attributed to the needs expressed by athletes or other influential individuals, rather than a clear appreciation of the full spectrum of the chiropractor’s clinical skillset. This issue has a tendency to result in chiropractors practicing in a limited capacity, often occupying the role of a manual therapist [11].

Moreover, and in relation to high-level elite level sporting events, chiropractors can also claim a role as service providers. However, this penetration is again, not due to a clear role and perceived value as part of the IPHCT, but rather as beneficiaries of a policy of athlete-centered service provision [11–13]. This situation is sub-optimal for the development of interprofessional practice, as the additional health outcomes benefit originates from a culture where the contributions of individual members are equally valued [4].

In Denmark, chiropractic has transitioned into a mainstream service provider with higher levels of mainstream integration being observed [14]. It is therefore conceivable that this general state of increased inclusion has manifested in the elite athletic health and performance practice. This context of practice has, however, not been explored.

Football is Denmark’s most popular sport with a documented 362,418 active football players in 2018 [15]. It is also the country’s wealthiest, with Denmark’s top league clubs currently carry a combined value of just over 186 million euros [16]. Moreover a recent audit of medical staff indicated that seven out of 17 teams (41.2%) associated with the Superliga [17], formally engage the service of a chiropractor. Given the economic capacity to employ an extended IPHCT and the presence of chiropractors in this context of care, an intriguing question arises: ‘Are chiropractors perceived as novelty practitioners, or is there evidence that the profession has begun to find traction as a member of the IPHCT with a defined role and perceived value?’

With the above in mind, the aim of this investigation was to explore the role and perceived value of chiropractors to Danish elite football clubs. And additionally, to explore barriers and facilitators to interprofessional practice involving chiropractors.

## Methods

### Study design

A comparative qualitative case study was conceptualized to explore a particular case of health care service provision [18].

### Theoretical stance

This design was underpinned by a constructivist stance, as we sought to understand the roles and consequent value of chiropractors by creating a meaningful co-construction of the experiences of participant stakeholders [19]. As both the primary researchers were chiropractors by profession, we considered how personal value systems might influence the research process. It was agreed that focus needed to be placed on a balanced (neutral) co-construction of meaning, especially in instances where data pointed towards negative attitudes towards the perceived value and roles of the chiropractor [20].

### Participants

In order to adequately observe this complex social action (the unit of analysis) adequately, we triangulated data from three units of observation; these being health care coordinators, chiropractors and athletes [21]. The study was limited to these data sources in order to focus feedback on insights relating to interprofessional practice, and also role and perceived value [22].

### Sampling

We used a maximum variation strategy to identify from the Danish Superliga organization, clubs that both included and excluded chiropractors as part of their healthcare team [23]. To achieve this, club websites were visited in order to retrieve healthcare coordinators or club administration contact information. A request for participation was then e-mailed to the coordinators. In the event that a positive response was received, the health care coordinators mediated access and eventual recruitment of athletes and chiropractors (where appropriate) [24].

In this study, eventual sample size depends on whether enough rich and thick data, has been procured so as to make further sampling meaningless (data saturation) [25]. To address this focus, we sought to include at least two instances, where chiropractors were utilized and two where they were not. Moreover, considering previous qualitative case study designs of this nature, a sample of approximately 10 respondents would suffice [7].

### Data collection

Participant perceptions and experiences were captured through individual interviews appropriate for making meaning of relevant social and personal life experiences [26]. Given the geographical spread of clubs, both face-to-face and telephonic interviews were conducted, to ensure sampling flexibility [27, 28].

Participants were provided with an information package before an interview was arranged and informed consent was sought from all participants before interviewing commenced.

Interviews were of the semi-structured variety, with interview guides consisting of open-ended questions designed to elicit responses around roles (functions) and values (benefits/impact) in general and more specifically barriers and facilitators to interprofessional practice [29]. All questions were supported by probes, to encourage further elaboration relevant to the responder groups.

Interviews were audio-recorded, transcribed verbatim into word text documents and anonymized. In this regard health care coordinators, athletes and chiropractors were assigned the acronyms ‘HC’, ‘AT’ and ‘CH’, respectively. The key quote positions in transcript was indicated with line numbers, designated with the prefix ‘L’. All key quotes were contextually translated into English to ensure that the semantic integrity of the text was maintained [30]. All data were stored on a password protected server and audio recordings were deleted upon the completion of the study.

### Data analysis

Data were analyzed using a modified thematic framework analysis approach [31], consisting of familiarization, identifying the thematic frame, indexing, charting and mapping/interpretation. To familiarize themselves with the data, two authors, JH and AK independently created a code book by inductively coding meaningful segments of text from the two initial interviews. By means of consulting researcher memos, code definition comparison and a consensus process mediated by the third author, CM, a common code book was then negotiated. This helped create an initial sense of the themes contained in the data. To further organize (index) the data, the rest of the interviews coded using a combination of deductive and inductive coding. Following the constant comparison method, as new codes emerged in subsequent interviews, either due to new observations or as a result of more abstract (axial) coding, the code book was amended and previous interviews scanned for new code presence [32]. Codes were subsequently further organized (charted) into code families, and a visual network created to abstract (map) the eventual themes.

Finally, a participant validation was performed [33], by asking four participants to affirm that the findings as reflective of the context of practice.

## Results

### Results of the sampling

**Fig. 1:**
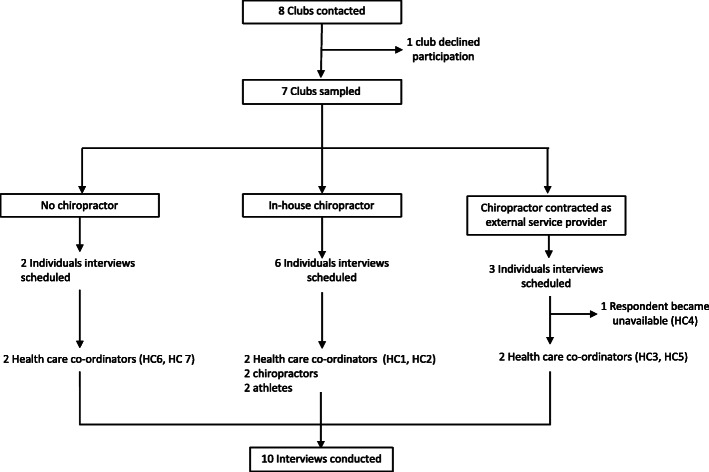
Flowchart of the sampling process

Ten individual interviews were conducted between 13th of September 2019 and 8th of November 2019, lasting on average 17 min per interview. Six were conducted at club houses, two at chiropractic clinics, one at a public café and one via telephone. As can be seen from Fig. 1, the sampling process identified instances with and without a chiropractor. However, a third category resulted as clubs employed chiropractors as in-house staff members and external health care providers. An “in-house therapists” was defined as having fixed weekly sessions at the site of the club, whereas to the “external service provider” functioned on an ad hoc basis. One health care coordinator (HC4) agreed to be interviewed but became unavailable for a face-to-face or telephonic interview during the data collection period. Interviews with chiropractors and athletes were conducted after interviews of the healthcare coordinator of the same clubs had been held.

### Thematic framework

**Fig. 2:**
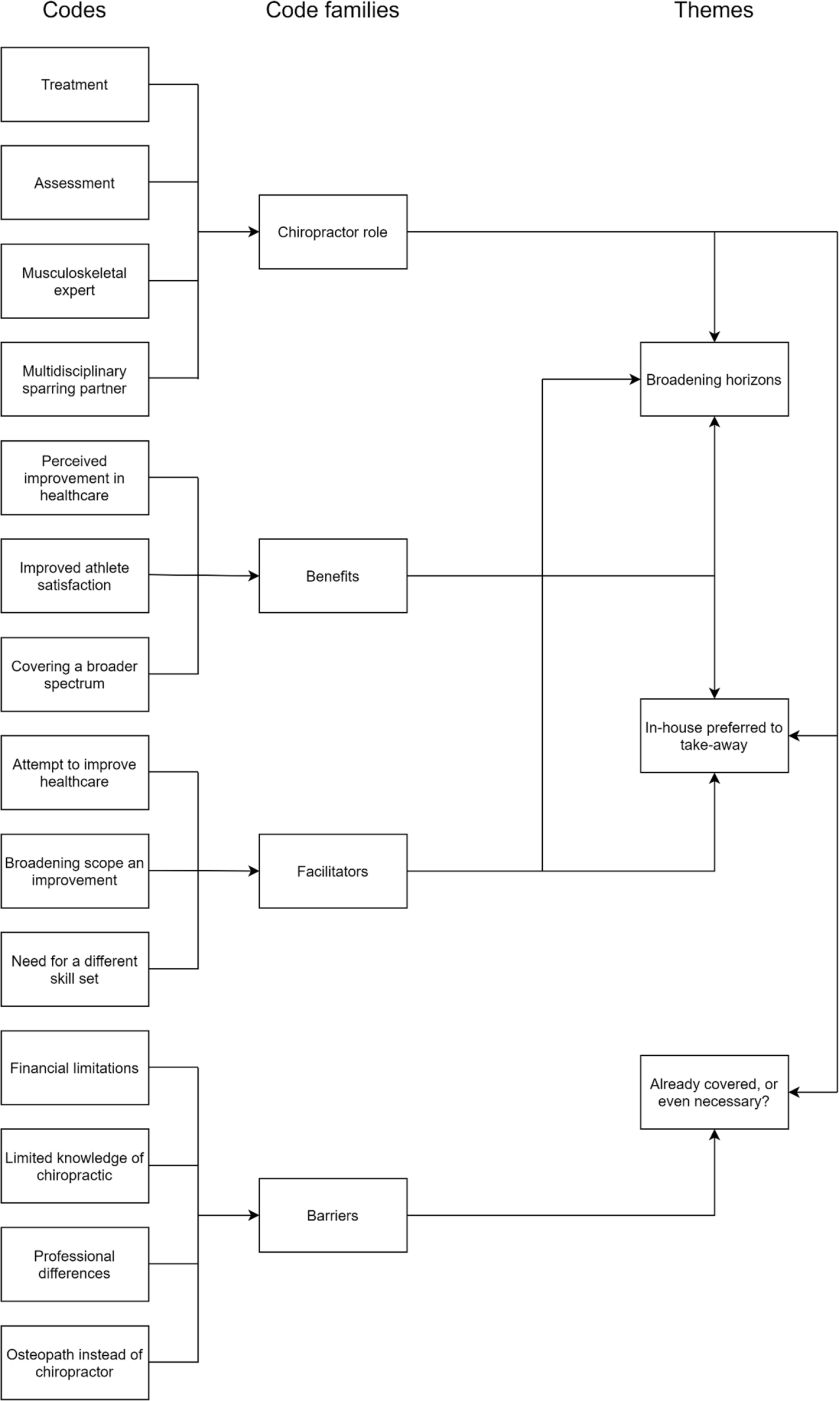
Organisation of data indicating emergence of themes

Based on participant responses, 14 individual codes were identified and organized into four code families, which included barriers and facilitators. From the codified data, 3 themes were abstracted, these being ‘broadening horizons’, ‘in-house preferred to take-away’ and ‘already covered or even necessary?’. The organization of the thematic framework is illustrated in Fig. 2.

### Broadening horizons

Generally speaking, health care coordinators engaged the chiropractor’s services with the aim of bolstering the expertise level around spine-related problems, but also to gain a different/new approach to assessment and treatment. According to HC 1 and 5:

I found that he would be able to complement me and the other physiotherapists. Particularly with the knowledge of the back and neck. I felt we needed to improve on that subject (HC1; L52-L54).

and

They of course have some special competence regarding manipulation. They have special skills in assessment of, for example, back patients regarding how a back moves, how an SI [sacro-iliac] joint moves and how you assess that… But they just have some other techniques and other approaches, that make it possible to cover the issue in the best way possible (HC5; L44-49).

A different approach was thought to bring other aspects to the pooled knowledge, enabling the IPHCT to make more informed decisions. According to HC1, 2 and 5:

The feedback from the players has been really good but also in the team, where we complement each other well, and something extra has been brought (HC1; L61-62)

Otherwise I would say the overall effect I have felt is that we have gotten an extra pair of hands and it is in regard to sparring and in multidisciplinary teamwork that they have contributed the most to me (HC2; L107-109).

and

I think there was a need for their knowledge and what they could do (HC5; L122).

Added value over previous service provision model was thought to lie in covering a broader spectrum of health care, resulting in a greater likelihood of pin pointing ‘the right diagnosis’ (HC5; L47–49). This discourse is highlighted by HC 2 and 3, respectively:

We cover everything, so nothing is left uncertain, because they [chiropractors] have a different sense and a different way of approaching the joints than I do. And it complements each other well, I think. I think we are covering a broader spectrum, also in treatment and I think that is where the gain is (HC2; L114-117).

and

I think it is important to cover a broad spectrum of what you do. We have two physios and a massage therapist now, … and then we send people externally, if we are missing something specific (HC3; L46-47).

Healthcare coordinators engaged chiropractors as spine-related musculoskeletal health experts offering specific discipline-specific knowledge regarding biomechanical injury mechanisms, patient assessment and diagnosis and competencies in conservative manual interventions such as manipulation, mobilization, and dry needling. Their rationale for adding the chiropractor is to broaden the shared pool of knowledge with the expressed benefit of better diagnoses and more comprehensive management.

### In-house preferred to take-away

The use of chiropractic services, at least initially, appeared to occur on an ad hoc basis. However, once established, the availability of the chiropractor became an issue for interprofessional practice, in particular effective care co-ordination. In this regard, consensus existed that contracting chiropractors as internal service provider, was a distinct improvement compared to earlier ad hoc utilization. This service provision evolution is illustrated in the following discourse from club 2:

… as time went by, I tried to see if we could improve, of course for the football club, but the chiropractors also wanted to contribute more. And then they started to come to the site of the club as well… it has evolved because that worked so well for us. We could make it, so the players were closer to treatment, …, that made it easier for the players and actually for the chiropractors as well, to keep control of things. Instead of seeing the players sometimes, and then not for 2-3 weeks (HC2; L39-47).

The interdisciplinary teamwork is good, especially in the later years where we have attended the training camps, …, that just makes us know each other’s strengths and weaknesses much better. … And it works with equality with respect for each other (CH2; L77-81),

I know that they will come tomorrow, so there he can crack my back and loosen my hip, then I maybe get the massage therapist to do that and that, so that I do not get double treatment… Planning wise it is also easy for us … That is nice (AT2; L32-37).

From a service delivery perspective, this developing relationship was perceived as beneficial to athletes as service delivery could more readily include curative and preventative care. In this regard HC 1 stated:

… the chiropractor also sees the same people repeatedly and not only when they get injured. Earlier it was only when they got injured. Let’s say they had an acute lumbago, then they would be sent to a chiropractor. Now he is more implemented every day in everything (HC1; L29-36).

A pragmatic benefit of having chiropractors as internal therapists was ‘getting extra pairs of hands’, so that there is ‘more time for [gaining] insight’ with each athlete (HC2; L102–104). This view was echoed by HC5, who saw shifting to in-house chiropractic services as a wish list item, stating: “We could use some extra hands and some extra time for insight” (HC5; L137–138).

For the chiropractor, attaining in-house service provider status provided a clear benefit of direct access to athletes. These benefits included athletes developing a better understanding of ‘what we [chiropractors] are good at and what the physio is good at ‘(CH2; L50–53). Moreover, care could be initiated timeously. In this regard CH1 observed:

… the previous physios were not fans of chiropractors; it was the players who needed to ask to be seen by a chiropractor … That was a problem because then we see the patient too late (CH1; L41-44).

Shifting chiropractors from external to in-house practitioners, provided healthcare coordinators with care continuity benefits. Moreover, having more practitioners available provided an additional advantage to individualized care strategies. And direct access to athletes provided the chiropractor with a better opportunity to timeously initiate appropriate care.

### Already covered, or even necessary?

In the two instances of clubs not utilizing chiropractors, the services deemed relevant to a chiropractor were instead assigned to an osteopath. In this regard HC6 and 7 stated:

… we have had osteopaths all along in the club instead of chiropractors… which you could argue on some points are similar (HC6; L6-10).

and

We do not have a chiropractor employed, but we have an osteopath employed and a part of the osteopath education is also chiropractic – at least in some ways with manipulations etc… (HC7; L2-3).

For HC7, the osteopath ‘only does manual therapy when he is with us’ (HC7; L32–33) and mainly with a preventative focus. In this regard HC6 and HC7 elucidated this role, stating:

… he [the osteopath] has no part in rehabilitation. The role he has is that some of the long-term injured players – some of them who are maybe back in training, but still have some irritations – I send them to him (HC7; L32-37).

The osteopath covers the preventive correction. What I see as the strength of the osteopath is to look at alignments, the whole person and where there are things that are …, badly compensated …, that we maybe can try to correct and see if it is compensated better before you start with for example strength training and all sorts of other things (HC6; L53-57).

Interestingly both respondents, despite their perceptions of chiropractors and osteopaths overlapping significantly, admitted to having a very limited knowledge of the chiropractors’ scope of practice. Specifically, HC6 and 7 stated:

But I will say, I know nothing about chiropractic (HC6; L72).

and

I do not know very much of the chiropractic education… I do not know the chiropractor’s role in rehabilitation and how good they are at that – and that is probably why it is physiotherapists we employ (HC7; L54-58).

From a practitioner utilization and cost-effectiveness perspective, HC7 argued that smaller clubs are better off with a lean healthcare team, rather than attempting to offer extended services. In this regard HC 7 argued that:

This is a club with not that much money… There is [only] money for two full time employees, and I prefer that instead of me being full time and four others sharing the other days, … And from those criteria I think it makes more sense to have two full time physiotherapists where one of them is an osteopath, than having a chiropractor employed (HC7; L14-19)

Finally, the seriousness of spinal problems as factors affecting training and performance in football were questioned and as a result the need for a practitioner with a limited spinal focus. For HC 7: “… we do not experience players who have an issue with their low back being out of training. That is very rare. That is a bit of an injury you must play through… Low back issues are rarely something that keeps our players out – max one to two days” (HC7; L42–44).

Chiropractors are not perceived as professionally unique and some health care coordinators share an affinity for other service providers groups that are perceived to provide a service equivalent to chiropractors. In instance of financial limitations coordinators may look towards a core provider team that may also be able to provide some of manual therapy options associated with chiropractic. The relevance of a professional group with a niche interest in the spine is questioned in the context of football.

### Barriers and facilitators

Based on respondent feedback, we identified four barriers and three facilitators relevant to chiropractor involvement in this context of practice (see also Table 1).

**Table 1:** Barriers and facilitators to chiropractor inclusion in inter-professional practice

Barrier	Key quote(s)	Facilitator	Key quote(s)
*Back pain expert relevance?*	… we do not experience players who have an issue with their low back being out of training. (HC7; L42–44).	*Shift toward extended health care delivery*	I found that he would be able to complement me and the other physiotherapists. Particularly with the knowledge of the back and neck (HC1; L52–54). We do not [decide] from our own subject knowledge. … we combine our subject knowledge and get a common output – and that is the output we release (HC3; L57–60).
*Uniqueness of chiropractor expertise*	… we have had osteopaths… instead of chiropractors… which you can say on some things are down the same road (HC6; L6–10).	*Perceived need for a (spinal) MSK expert*	I think there was a need for their [chiropractor] knowledge and what they could do (HC5; L122).
*Existing provider group competition*	… the previous physios were not fans of chiropractors, it was the players who needed to ask to be seen by a chiropractor… (CH1; L41–44). I do not know the chiropractor’s role in rehabilitation and how good they are at that – and that is probably why it is physiotherapists we employ (HC7; L54–58).	*Athlete satisfaction*	If he was not here and we could not use him, it would be a shortcoming, … (AT1; L60–62) We benefit much from them… The chiropractors are more long-term solution oriented. I think that is the way so many utilize them… (AT2; L20–26)
*Financial limitations*	It is not that I do not want to be there more, but I do not think the club can afford it (CH1; L99–101). There is [only] money for two full time employees and I prefer that instead of me being full time and four others sharing the other days, to be as few as possible. And from those criteria I think it makes more sense to have two full time physiotherapists… (HC7; L14–19).		

When not included as service providers, we observed two barriers relating to the expertise chiropractors are perceived to offer. Firstly, and in a general sense, back pain was perceived as self-remitting. And as a consequence, the need for a back pain expert was questioned. Secondly, the uniqueness of chiropractors as providers of manual therapy interventions was queried, with occupational groups. Specifically, osteopaths were perceived to have similar utility and were suggested as an alternative.

Professional groups, in this instance physiotherapists, appear to compete with chiropractors for positions as in-house service providers.

Budgetary limitations, again in a general sense, appear to reduce the level of chiropractor service utilization. However, perhaps more importantly, it would appear that with a tight health care budget, the coordinator is likely to create a lean health care team, composed of providers with known roles.

With regards to facilitators, health care coordinators incorporating chiropractic services, endorsed the notion of the interprofessional team, offering a superior health care solution, due to a larger pool of shared knowledge. Moreover, and with a focus on back and neck-related problems, chiropractors were specifically seen as a provider group with an important contribution to offer. Lastly, the utility of chiropractors as part of the in-house provider team, was strongly endorsed by athlete responders. This perspective appears grounded in the need for regular contact with chiropractors to manage long-term health care issues.

## Discussion

### Role

Health care coordinators expressly engaged in-house chiropractors as musculoskeletal health experts offering discipline-specific knowledge and with a role in clarifying biomechanical injury mechanisms, undertaking patient assessment and diagnosis and providing a variety of conservative manual interventions. In this regard, our results differ from previously reported data, and in particularly those of Therberge [11], who reported the engagement of chiropractors in a limited role and based on pressure created by athletes.

However, this role designation stood in contrast with clubs not engaging the services of a chiropractor. In these instances, osteopaths were instead utilized in a specific therapist role providing a particular manual intervention and were excluded from the planning and implementation of rehabilitation protocols. Perhaps somewhat unsurprising, chiropractors were not perceived as having a unique professional role. This finding is more in-tune with previous studies describing the designated role of chiropractors functioning as a part of health care teams [11–13].

### Value

The perceived value chiropractors as part of the in-house health care team, lay in the broadening of the shared pool of knowledge, yielding better diagnoses and more comprehensive management strategies. Theoretically speaking, this perceived value relates to the notion of cognitive maps, where a multidisciplinary cognitive map offered by the IPHCT, is more efficient than a monodisciplinary one (the single practitioner), for developing accurate, strong problem-solving routines [34].

A novel observation and operationalization of added value, emerged from instances, where chiropractors transitioned from external to in-house practitioners. In particular, with direct access to athletes, the chiropractor was able to initiate appropriate care in a timely manner, and in this way better maintain individualized care strategies. This improved care continuity is in-tune with the athlete-centered focus to care considered desirable in this practice context [6, 35].

### Salient issues

A key perceived driver for the development of interprofessional teams is strategic organisational policy [34]. In this regard, our data is suggestive of the increased involvement of chiropractors within more affluent clubs. However, interprofessional practice is also thought to thrive in collaboration-rich settings [3, 4]. From the responder feedback, the decision to engage in-house chiropractors was based on the evolution of successful collaborative practice or a combination of these factors. Our data did not elucidate the issue of strategic health care policy.

Our data suggests, that chiropractors have claimed a role as musculoskeletal health expert in Superliga clubs. However, it was not possible to discern, whether this role was generalized or limited to spinal complaints. It stands to reason that with a generalized role, the chiropractor is more likely to achieve core member status, rather than remaining a value-added service provider.

### Strengths and limitations

Our sampling process allowed for the observation of contrasting cases; the third sampling category, that of the externally contracted chiropractor was particularly useful in clarifying the developing practice role of chiropractors.

The role of osteopaths as service providers was only observed from the perspective of health care coordinators. A thicker description and triangulation with osteopaths would have provided greater saturation.

## Conclusion

When functioning as in-house team members, chiropractors are engaged in interprofessional practice characterized by meaningful collaboration. Their presence is perceived to improve the quality of athletic health care through the addition of discipline-specific knowledge, diagnostic triage capabilities and increased intervention options. In instances where chiropractors are not utilized, the role of the spinal health expert appears to be limited to that of a technician/therapist. It is, however, unclear whether chiropractors can claim core team member status. Further exploration of this interesting context of interprofessional practice is warranted.

## Data Availability

Interview transcripts are available on request.
